# Society Proceedings

**Published:** 1895-07

**Authors:** 


### SOCIETY PROCEEDINGS.

---

### MISSOURI VALLEY VETERINARY ASSOCIATION.

THE second annual meeting of this Association was held in Leavenworth, Kansas, at the National Hotel, June 12th. There were present Drs. Hunter and Ernst, Leavenworth; Netherton, Gallatin; Netherton, Carrollton; Biart, Lansing; Harrison, Atchison; Sihler, Stewart, Airth, and Barth, Kansas City, Kans.; Thompson, Olathe; Topping, Paola; Hill, Bray, and Wattles, Kansas City, Mo.

Officers for coming year elected were: *President*, Stewart; *First Vice-President*, Harrison; *Second Vice-President*, Netherton (G. T.); *Secretary-Treasurer*, Hunter; *Censors*, Bray, Hill, Wattles, Sihler, Barth.

Papers were read by Harrison on "Epizootic Abortion in Cattle;" Barth on "Actinomycosis;" Sihler on "Fever;" Ernst on "A Selection of Cattle to Prevent Disease in the Human Family;" and Stewart on "The Various Tape-Worms of Man and Animals."

The following resolutions were passed: That there does not exist at the present time, and has never existed, any pleuro-pneumonia contagiosa among cattle in the State of Kansas. That it is placing the health of the general public in danger to appoint any but graduates of veterinary colleges as inspectors of meat, milk, and dairies, and that we recommend that at least one veterinarian be appointed upon each board of public health.

The meeting will be held in Kansas City, Mo., during carnival week.

J. H. WATTLES,
Reporter.

---

### NEW YORK STATE VETERINARY MEDICAL SOCIETY.

AT the special meeting held in the Lecture-room of the New York College of Veterinary Surgeons, New York City, on June 11, 1895, the roll-call showed a number of veterinary surgeons present. The reading of the minutes of the previous meeting was dispensed with. The President, Dr. R. S. Huidekoper, explained in a few remarks the reasons for calling this special meeting. He said that it was in order to comply with a clause in the bill (recently become a law) wherein the New York State Veterinary Medical Society is required to nominate ten of its members, whose names are to be transmitted to the Board of Regents, who, according to law, are to appoint five of the ten to act as the State Board of Veterinary Examiners, to hold office for five years.

The following are the ten members who were chosen by ballot of the members present, to be submitted to the Board of Regents from which to make their appointments: Drs. R. S. Huidekoper, Nelson P. Hinkley, Claude D. Morris, William Henry Kelly, John Wende, W. L. Baker, Arthur O'Shea, John A. Bell, J. A. Hanson, and Prof. James Law.

There was also a resolution offered giving notice to the Society that at the next annual meeting there would be a resolution offered to amend the by-laws so as to read that all legally-registered veterinarians in the State of New York be eligible to present their applications to become members of the New York State Veterinary Medical Society. This resolution was accepted and ordered spread on the minutes.

The meeting then adjourned until the next annual meeting, which will be held in the Lecture-room of the New York College of Veterinary Surgeons, No. 154 East Fifty-seventh Street, New York City, September 5 and 6, 1895.

NELSON P. HINKLEY,
Secretary.

### NEW HAMPSHIRE VETERINARY MEDICAL ASSOCIATION.

A SPECIAL meeting took place in Concord, at the Eagle Hotel, on June 13th. Dr. Lilico's name was voted upon favorably for membership. A paper was read by Dr. Pope on "The Diagnosis of Tuberculosis," and a discussion followed. Adjourned until the first Tuesday in October.

L. POPE, JR.,
Secretary.

### CALIFORNIA STATE VETERINARY MEDICAL ASSOCIATION.

A MEETING of the California State Veterinary Medical Association was held at the Commercial Hotel, Stockton, Cal., June 12, 1895.

The meeting was called to order by the President, Dr. C. B. Orvis, at 2 P.M.

Upon roll-call the following members responded: Drs. Orvis, Eddy, Fox, Spencer, Sr.; Lemke, Williams, Hogarty, and Archibald. Visitor, Dr. Eddy, Sr.

Letters of regret were read from absent members.

The minutes of the previous meeting were read and approved.

The Board of Examiners reported favorably on the application of C. L. Megowan, whereupon, on motion, C. L. Megowan was unanimously elected to membership.

Under the head of unfinished business, the election of a delegate to the next meeting of the National Organization was taken up, with the result that Dr. R. A. Archibald was unanimously elected to represent the Association at the next meeting of said organization.

Owing to the fact that Dr. Archibald was elected a delegate, he refused to collect the assessment levied at the last meeting for the purpose of defraying the delegate's expenses.

Dr. D. F. Fox was appointed to collect the assessment.

Under the head of reading of papers, etc., and discussions, Dr. Lemke was called upon to entertain the meeting, which he did by giving a review of the work he was doing toward the prophylactic treatment of anthrax in the central portion of the State, under the direction of the Pasteur Anthrax Vaccine Company. He stated that the above-named company had employed him for the purpose of determining to what extent anthrax prevailed in California, with a view of commencing a warfare on the disease by means of vaccination. He said he desired the co-operation of every practitioner in the State, more especially the members of the Association. He further stated that he had investigated the disease to a considerable extent, and he had about come to the conclusion that there was a difference between the disease and true anthrax, the mortality was not so great. He wished to get an expression from the members present, who had had some experience with the disease, as to their opinion of the nature of the disease. He was unwilling to vaccinate until he was thoroughly convinced of its true nature. He requested the members to prepare specimens of animals that had died from the disease, and forward them to him, so he could have them submitted to a microscopical examination. He went on to give some statistics and data regarding the loss of live stock, the financial loss, and the mortality of the disease.

Considerable discussion followed Dr. Lemke's remarks, and the Doctor was subjected to a rigid cross-examination by most of the members present, all of whom expressed a willingness to assist him by every means in their power.

The next event on the programme was the reading of a very instructive paper on "Bronchitis," by Dr. D. F. Fox, who described the different phases of the disease and the different methods of treating same.

Dr. J. H. Eddy followed with an excellent and carefully prepared paper on "The Circumstances which Modify the Action of Medicine." Both subjects were thoroughly discussed by the members.

On motion by Dr. Spencer, a vote of thanks was tendered the essayists for the able and masterly manner in which they had entertained the meeting.

Under the head of new business, the Chair appointed the following-named gentlemen as essayists for the next meeting: Drs. Spencer, Jr.: Skaife, Pierce, and Shaw.

Dr. R. A. Archibald submitted the following resolution:

*Whereas*, The live-stock interests of this State are suffering from the malignant influences of contagious and infectious diseases; and

*Whereas*, The health of the public of this great State is also jeopardized by the presence of said contagious and infectious diseases which affect the domestic animal; and

*Whereas*, We, the members of the California State Veterinary Medical Association, realizing that this state of affairs is wholly due to the fact that there are no laws on the statute books of this State sufficiently adequate to control the ravages of these contagious and infectious diseases; therefore, be it

*Resolved*, That the President be and he is hereby authorized and requested to appoint at the next meeting of the Association, which will be held at Sacramento, a committee consisting of three members, whose duty it shall be to wait on or communicate with the Governor of this State, with a view of prevailing upon him to appoint a commission, to consist of one veterinarian, one physician, one lawyer, one dairyman, and one stockman, who shall receive no compensation, and whose duty it shall be to devise ways and means whereby the public health and the live-stock interests of this State may best be protected from the ravages of contagious and infectious diseases, such as anthrax, tuberculosis, glanders, hog-cholera, etc.

Considerable discussion followed the presentation of the resolution, and the author was highly complimented for his ingenuity in drafting same.

Upon motion of Dr. Spencer, Sr., the resolution was unanimously adopted.

There being no further business to come before the meeting, on motion by Dr. Fox, the by-laws were suspended, and the meeting adjourned to meet in Sacramento on Tuesday, September 10, 1895.

On Thursday, June 13, 1895, the members of the California State Veterinary Medical Association assembled, by invitation of Dr. C. B. Orvis, at his infirmary at 258 Lafayette Street, Stockton, to participate in a clinical entertainment.

The first operation on the programme was the reduction of a ventral hernia by Dr. H. A. Spencer, assisted by Dr. Archibald. The operation was the radical one for the cure of a hernia, and was performed under strictly antiseptic and aseptic rules.

The next operation was performed by Dr. Lemke. This was the case of an old, long-standing fracture of the scapula at its upper third, from which a fistula resulted which necessitated the trephining of the scapula.

Other operations of minor importance followed, closing the entertainment, which was voted an entire success by the members present.

R. A. ARCHIBALD,
Secretary.

---

### SCHUYLKILL VALLEY VETERINARY MEDICAL ASSOCIATION.

THE annual meeting of the Schuylkill Valley Veterinary Medical Association was held at the office of Drs. Sallade and Fegley, Pottsville, Pa., June 20th. The election of officers resulted in the re-election of *President*, S. G. Burkholder, Denver; *Vice-President*, W. H. Moyer, Elizabethville; *Recording Secretary*, U. G. Frederici, Tamaqua; *Treasurer*, F. H. McCarthy, Ashland. Otto Noack, of Reading, was elected *Corresponding Secretary*. *Trustees*, Otto Noack, Reading; Eli Snyder, Orwigsburg; and J. C. Faughnan, Shamokin.

After the reading of the minutes of the preceding meeting, N. K. Fegley, graduate of the National Veterinary College, and Bert Hagenbuch, graduate of the Veterinary Department, University of Pennsylvania, were proposed for membership and duly elected.

Among the papers read was one on "Meat and Milk Inspection," by Dr. Otto Noack. Dr. Sallade spoke on the importance to the profession of every veterinarian becoming an educator in his community; how important it was that all should combine their efforts in the direction of extolling their own importance; that by such efforts much had already been accomplished and legislation secured which placed the profession in a much better light than it was in when he entered it; and that it was the duty of every young veterinarian who entered the field at the time when the path had been paved for him to labor intelligently to advance the profession. He advised him to join farmers', dairymen, and horseshoers' associations, and to take part in the discussions, which would afford him opportunities to display his superior knowledge, and thus advance steadily in the minds of his clients not only individual interest, but the interest of the profession at large, as well as the interest of the community, etc. He then read a paper on "Stable Hygiene," once produced at a farmers' institute, and it was agreed to devote due attention to this kind of work.

Drs. Sallade, Noack, and Frederici were appointed a committee to draft an address or memorial to all borough and city councils comprised in the territory occupied by the Society, asking for legislation on milk and meat inspection, and the importance of having our profession represented on the boards of health, etc.

Dr. Fegley was appointed essayist; subject, "Rabies."

Association to meet again in three months at same place, when committees will be announced by President Burkholder.

---

### GERMAN VETERINARY MEDICAL ASSOCIATION OF NEW YORK AND VICINITY.

THE general and annual meeting of the Society was held Tuesday, April 16, 1894, at Dr. Sattler's Hospital, Newark.

The meeting was called to order by the President, Dr. L. R. Sattler, Newark. Upon roll-call the following gentlemen responded to their names: Drs. Turner, Ogden, Leis, Aurker, Wellner, Serling.

The minutes of the previous meeting were read and approved.

Several communications from absent members were read by the Secretary, expressing regrets for being unable to attend the meeting. The Secretary's report was read, which showed the Association to be in a flourishing condition numerically, and the roll-call showed a large increase in membership.

"Three years ago this Society was reorganized, and I was chosen as your Secretary, and you have seen fit at every election of officers since that time to honor me with a re-election; a trust that I have not only felt honored and pleased with, but one that I have faithfully and earnestly tried to fulfil. At every preceding report I have had encouraging and gratifying results of the condition of the Association to submit for your consideration; the record of the past year is no exception, and I am sure that you will not contradict me when I say that our Society ranks on equal plane with any veterinary organization in the States. We have good reason to congratulate ourselves on our success, and I am positive that not a single member of our Society to-day will say that he has not received some benefit from the pleasant and interesting meetings of this Association, as the Association's labors are not matters of dollars and cents or individual benefit, but are for the advancement of the veterinary profession and the protection of the public in the State of New York.

"During the past years our monthly meetings have been successful and well attended. Interesting papers were read and well received, and no doubt brought forth good fruit. At the meeting on Tuesday, November 13, 1893, we were honored by the presence of our honorary member, Prof. Dr. A. Liautard, who, by his vast experience, rendered us great and valuable aid in the important work of making the meetings interesting. At that meeting he read a paper, 'Experimental Researches upon the Use of Malleine.'

"A deplorable fact to which I wish to call your attention is the death of two of our best members, Dr. A. Kuntz, the founder of this Association, and Dr. Robert Leis. Both were members in good standing. They will never be forgotten by us.

"Our financial condition will be reported by our Treasurer, Dr. J. Turner, and I trust that my accounts and vouchers, which are here for your inspection, will be found correct and satisfactory. In closing my report I wish to thank most sincerely the JOURNAL OF COMPARATIVE MEDICINE and the *Veterinary Review* for their very generous act of kindness to the Society for fully publishing all our notices and proceedings. Also to thank the members individually and the officers for their kind assistance and consideration during the last year, and for your kind attention to my present report."

Dr. Serling: I move the report be accepted and placed on file. Seconded by Dr. Leis. Carried.

Then the report read by the Treasurer showed the Association to be in a rather straitened condition financially.

The Chair declared now that nominations for officers for the ensuing year were in order. The election took place and all officers were re-elected unanimously. The Secretary was requested to announce the result of the election, which he did, as follows: *President*, Dr. L. R. Sattler, of Newark; *Vice-President*, Dr. Rudolph Leis, of Newark; *Secretary*, Dr. Hermann Wellner, of New York; *Treasurer*, Dr. Fred. Turner, of New York; *Board of Censors*, Drs. Serling, Simmon, and Ogden.

The newly-elected President upon taking his seat made a few appropriate and well-chosen remarks. He thanked the members for the great honor they had done him, and promised to do all in his power to make the meetings interesting and instructive, and in that way further the interests of the Association. He deplored the lack of attendance at the meetings, and said he would leave no stone unturned to bring the members together and have them work hand-in-hand for the advancement of the profession in this great and glorious State.

Then the motion that the Society should be incorporated was accepted.

Dr. Sattler performed then, with the assistance of the present members, an interesting operation, sectio-cæsaria, on a bull-terrier bitch, three years old. He brought forth six puppies, two alive and four dead ones. At the suggestion of Dr. Serling the two puppies were put in an incubator, and after three-quarters of an hour they were able to drink milk from the bottle.

Dr. Wellner reported a case of glanders, where a difference of opinion had been expressed by Dr. Corlies, of Newark; the lesions of the head were shown to be the results of glanders; the microscopical and bacteriological experiments, which he had done with the aid of Dr. Sattler, also corroborated the diagnosis.

There being no further business before the meeting it adjourned to meet at Hoboken, Myers's Hotel, May 14, 1895.

H. WELLNER, D.V.S.,
Secretary.

